# Journal of Biomedical Science Reviewer Acknowledgement 2015

**DOI:** 10.1186/s12929-016-0243-6

**Published:** 2016-02-24

**Authors:** Wen-Chang Chang

**Affiliations:** Taipei Medical University, Taipei, Taiwan

## Abstract

The editors of *Journal of Biomedical Science* would like to thank all our reviewers who have contributed to the journal in Volume 22 (2015).

**L Adorini**

USA

**A Ahmad**

Canada

**Miho Akimoto**

Japan

**Ola Ali**

Egypt

**Jalal Alizadeh**

Austria

**Paulo Amaral**

UK

**Doblin Anak Sandai**

Malaysia

**Katsuyuki Aozasa**

Japan

**Nadezda Apostolova**

Spain

**Bahram Arjmandi**

USA

**N Azevedo**

Portugal

**Li-Yuan Bai**

Taiwan

**Andrey Barkhash**

Russia

**S Barr**

Canada

**F Barreto dos Santos**

Brazil

**Alexander Bayden**

USA

**Andrea Belin**

Sweden

**Thorsten Berger**

Canada

**Ben Berkhout**

Netherlands

**Robbert Berkhout**

Netherlands

**Claudia Biondi**

Argentina

**Ernst Bomhard**

Germany

**Emanuela Bostjancic**

Slovenia

**A Bretscher**

USA

**Giacomina Brunetti**

Italy

**Chandana Buddhala**

USA

**D Canals**

USA

**A Carnero**

Spain

**Tai-Lung Cha**

Taiwan

**D Chan**

Hong Kong

**Julie Y.H. Chan**

Taiwan

**Nei-Li Chan**

Taiwan

**Samuel H.H. Chan**

Taiwan

**Kai-Ping Chang**

Taiwan

**Nan-Shan Chang**

Taiwan

**Jer-Wei Chang**

Taiwan

**Jang-Yang Chang**

Taiwan

**Long-Sen Chang**

Taiwan

**Ming-Fu Chang**

Taiwan

**Rui Chang**

USA

**Jang-Yang Chang**

Taiwan

**Tsuchung Chang**

Taiwan

**Wei-Chiao Chang**

Taiwan

**Yu-Sun Chang**

Taiwan

**Zee-Fen Chang**

Taiwan

**Lee-Young Chau**

Taiwan

**Wassim Chehadeh**

Kuwait

**Yi-Jen Chen**

Taiwan

**Bing-Chang Chen**

Taiwan

**Ben-Kuen Chen**

Taiwan

**Chien-Chang Chen**

Taiwan

**Chang-Han Chen**

Taiwan

**Jau-Nian Chen**

USA

**Yung-Ming Chen**

Taiwan

**Ching-Chow Chen**

Taiwan

**Guobing Chen**

USA

**Kuen-Feng Chen**

Taiwan

**Pei-Jer Chen**

Taiwan

**Ruey-Hwa Chen**

Taiwan

**Chang-Han Chen**

Taiwan

**Ya-Wen Chen**

Taiwan

**Yi-Rong Chen**

Taiwan

**Wei-Yu Chen**

Taiwan

**Yen-Chou Chen**

Taiwan

**Yi-Jen Chen**

Taiwan

**Yi-Rong Chen**

Taiwan

**I Cherepanova**

USA

**Ya-Hui Chi**

Taiwan

**Ing-Ming Chiu**

Taiwan

**Jeng-Jiann Chiu**

Taiwan

**Il Kyu Cho**

USA

**S Choi**

Korea, South

**Yu-Ting Chou**

Taiwan

**Sin-Tak Chu**

Taiwan

**Lee-Ming Chuang**

Taiwan

**PC Chuang**

Taiwan

**Shuang-En Chuang**

Taiwan

**Huai-hu Chuang**

Taiwan

**Yao-Chung Chuang**

Taiwan

**Yung-Jen Chuang**

Taiwan

**R Chun**

USA

**Bon-chu Chung**

Taiwan

**Chih-Pin Chuu**

Taiwan

**Cristina Clement**

USA

**Clementina Cocuzza**

Italy

**Dawn Coletta**

USA

**G Coombs**

Australia

**Clark Cullen**

USA

**Guoli Dai**

USA

**Niann-Tzyy Dai**

Taiwan

**P Debre**

France

**Jeffrey Deiuliis**

USA

**C Dissous**

France

**Sipho Dlamini**

South Africa

**W Duan**

USA

**E Eugenin**

USA

**Hua-Chang Fang**

Taiwan

**Kang Fang**

Taiwan

**Kleber Farias**

Brazil

**P Fei**

USA

**Gregg Fields**

USA

**Jorge Filmus**

Canada

**Janet Flatley**

UK

**J Frisch**

Germany

**Wen-Mei Fu**

Taiwan

**Bruno Gaillet**

Canada

**David Gewirtz**

USA

**Payam Gharibani**

USA

**Anuradha Ghosh**

USA

**Tommaso Giani**

Italy

**Stephen Ginsberg**

USA

**N Gleicher**

USA

**M Golczak**

USA

**M Gotte**

Germany

**M Gottschalk**

Canada

**J Greenberger**

USA

**R Guabirada**

UK

**Ana Paula Guedes Frazzon**

Brazil

**Jih-Hwa Guh**

Taiwan

**K Gurvinder**

Australia

**K Gurvinder**

Australia

**B Haagmans**

Netherlands

**Charles Halsted**

USA

**Stephen Hare**

UK

**John Harwood**

UK

**L Hathaway**

Switzerland

**M Henry-Stanley**

USA

**Edward Hitti**

Saudi Arabia

**S Hladky**

UK

**Tingjun Hou**

China

**George Hsiao**

Taiwan

**Pei-Wen Hsiao**

Taiwan

**Chung-Bao Hsieh**

Taiwan

**Hsiu Mei Hsieh**

Taiwan

**Patrick Hsieh**

Taiwan

**Po-Shiuan Hsieh**

Taiwan

**Sung-Tsang Hsieh**

Taiwan

**Chin Hsu**

Taiwan

**Hsin-Ling Hsu**

Taiwan

**Ching-Sheng Hsu**

Taiwan

**Kuei-Sen Hsu**

Taiwan

**Yu-Juei Hsu**

Taiwan

**Hsin-Ling Hsu**

Taiwan

**Yi-Ping Hsueh**

Taiwan

**Teh-Min Hu**

Taiwan

**Eagle Yi-Kung Huang**

Taiwan

**F Huang**

USA

**Chung-Feng Huang**

Taiwan

**Yi-Tsau Huang**

Taiwan

**Jing-Ding Huang**

Taiwan

**Kun-Lun Huang**

Taiwan

**Li-Rung Huang**

Taiwan

**Shih-Ming Huang**

Taiwan

**Tze-Sing Huang**

Taiwan

**Tur-Fu Huang**

Taiwan

**Menggui Huang**

USA

**Xupei Huang**

USA

**Y Huang**

Taiwan

**Yishuian Huang**

Taiwan

**Ying-Zu Huang**

Taiwan

**Dueng-Yuan Hueng**

Taiwan

**Wen-Chun Hung**

Taiwan

**Li-Man Hung**

Taiwan

**Yi-Jen Hung**

Taiwan

**Liang-Yi Hung**

Taiwan

**Gero Hutter**

Germany

**John Imig**

USA

**Nobue Itasaki**

UK

**Jukka Jernvall**

Finland

**Lan Jiang**

USA

**Shih-Sheng Jiang**

USA

**Zheng Jiang**

USA

**Jeffrey Jones**

USA

**Yuh-Shan Jou**

Taiwan

**Chi-Chang Juan**

Taiwan

**Suh-Hang Juo**

Taiwan

**S Jurriaans**

Netherlands

**Shinji Kamada**

Japan

**S Kamiya**

Japan

**J Kanczler**

UK

**J Kang**

Korea, South

**Reiji Kannagi**

Taiwan

**Jia-Horng Kao**

Taiwan

**Murat Kasap**

Turkey

**S Kent**

Australia

**H Kida**

Japan

**Ushio Kikkawa**

Japan

**K Kim**

USA

**M Knight**

USA

**Jan Korbel**

Germany

**John Kung**

Taiwan

**Jean-Cheng Kuo**

Taiwan

**Yu-Min Kuo**

Taiwan

**Ching-Chuan Kuo**

Taiwan

**Kuo-wang Tsai**

Taiwan

**Dortet Laurent**

France

**Hsuan-Shu Lee**

Taiwan

**T-C Lee**

Taiwan

**Wei-Chia Lee**

Taiwan

**Huei Lee**

Taiwan

**Hwan Young Lee**

Korea, South

**Ming-Shyue Lee**

Taiwan

**Shou-Dong Lee**

Taiwan

**Hsin-Chen Lee**

Taiwan

**Yi-Hsuan Lee**

Taiwan

**Tzong-Shyuan Lee**

Taiwan

**Wen-Sen Lee**

Taiwan

**Yi-Hsuan Lee**

Taiwan

**Yi-Chia Lee**

Taiwan

**Guey-Jen Lee-Chen**

Taiwan

**Steve Leu**

Taiwan

**Euphemia Leung**

New Zealand

**C Li**

Taiwan

**Quanhai Li**

China

**Hua-Jung Li**

Taiwan

**Chih-Chia Liang**

Taiwan

**Shu-Mei Liang**

Taiwan

**Chun-Yen Lin**

Taiwan

**Feng-Yen Lin**

Taiwan

**Kwang-Huei Lin**

Taiwan

**Kai-Ti Lin**

Taiwan

**Kuo-I Lin**

Taiwan

**Shuei-Liong Lin**

Taiwan

**Ya-Wen Lin**

Taiwan

**Jen-der Lin**

Taiwan

**Kai-Ti Lin**

Taiwan

**ShuWha Lin**

Taiwan

**Su-Fang Lin**

Taiwan

**Yi-Ling Lin**

Taiwan

**Wen-Chang Lin**

Taiwan

**Wan-Wan Lin**

Taiwan

**Yee-Shin Lin**

Taiwan

**M Linnebank**

Germany

**Jyh-Ming Liou**

Taiwan

**Jun-Yang Liou**

Taiwan

**Hsiao-Sheng Liu**

Taiwan

**Chun-Jen Liu**

Taiwan

**Fu-Tong Liu**

USA

**Chen-Hua Liu**

Taiwan

**Wang-Ta Liu**

Taiwan

**Shing-Hwa Liu**

Taiwan

**Shih-Tung Liu**

Taiwan

**Shing-Hwa Liu**

Taiwan

**Ko-Jiunn Liu**

Taiwan

**Y Liu**

USA

**Z Liu**

USA

**Jeng-Fan Lo**

Taiwan

**J Louboutin**

Jamaica

**Sheng-Nan Lu**

Taiwan

**Shao-Chun Lu**

Taiwan

**Chih-Cherng Lu**

Taiwan

**Ida Gjervold Lunde**

USA

**Marthandan Mahalingam**

USA

**M Marazzi**

Italy

**T. Masami**

Japan

**Isao Matsuura**

Taiwan

**N Micale**

Italy

**Abhisek Mitra**

USA

**Jigar Modi**

USA

**David Morris**

USA

**P Muller**

Switzerland

**E Nakayama**

Japan

**Britto Nathan**

USA

**Melyssa Negri**

Brazil

**A Niaj**

Poland

**B Nikolajczyk**

USA

**N Nunthaboot**

Thailand

**Chunliu Pan**

China

**Liuliu Pan**

USA

**Li-Heng Pao**

Taiwan

**J Park**

Korea, South

**M Parsons**

Australia

**S Pechine**

France

**Hwei-Ling Peng**

Taiwan

**A Perl**

USA

**Peter Bradding**

UK

**A Ping**

USA

**John Pitman**

USA

**Hemant Poudyal**

Japan

**Spyros Pournaras**

Greece

**Howard Prentice**

USA

**Prabhakar Putheti**

USA

**M Rashid**

Pakistan

**Mickae Rialland**

France

**Gabriel Rinaldi**

UK

**Harrison Roger**

USA

**J Routy**

Canada

**U Ruffing**

Germany

**Chiranjeevi Sandi**

UK

**Michael Schmidt**

USA

**Erhard Seifried**

Germany

**Chia-Ning Shen**

Taiwan

**Ethan Shevach**

USA

**Sheau-Yann Shieh**

Taiwan

**Jin-Yuan Shih**

Taiwan

**Shin-Ru Shih**

Taiwan

**Venkatakrishna Shyamala**

India

**Jia-Fwu Shyu**

Taiwan

**Kou-Gi Shyu**

Taiwan

**Rajvir Singh**

USA

**Po-Chi Soo**

Taiwan

**K Steinmetzer**

Germany

**Tung-Hung Su**

Taiwan

**Huaichang Sun**

China

**Cheuk-Kwan Sun**

Taiwan

**Nao Suzuki**

Japan

**Man-Sun Sy**

USA

**Ming-Hong Tai**

Taiwan

**You-Lin Tain**

Taiwan

**Xiaoyu Tain**

Hong Kong

**Pao-Luh Tao**

Taiwan

**Rui Tao**

USA

**Woan-Yuh Tarn**

Taiwan

**Omid Teymournejad**

Iran

**Chenxi Tian**

USA

**N Topley**

UK

**Kenneth Tsai**

USA

**Kun-Chih Tsai**

Taiwan

**Hsien-Chun Tseng**

Taiwan

**Che-Se Tung**

Taiwan

**Alexandar Tzankov**

Switzerland

**Natsuo Ueda**

Japan

**Haluk Vahaboglu**

Turkey

**M Varecha**

Czech Republic

**E Vicenzi**

Italy

**KS Vishwanatha**

USA

**Hsian-Jenn Wang**

Taiwan

**Jaw-Yuan Wang**

Taiwan

**Jia-Yi Wang**

Taiwan

**Danny Ling Wang**

Taiwan

**Lu-Hai Wang**

Taiwan

**Ju-Ming Wang**

Taiwan

**Feng-Sheng Wang**

Taiwan

**Jin-Town Wang**

Taiwan

**Feng-Sheng Wang**

Taiwan

**Jin-Town Wang**

Taiwan

**Ju-Ming Wang**

Taiwan

**Yi-Ching Wang**

Taiwan

**Yun Wang**

Taiwan

**Yau-Huei Wei**

Taiwan

**Jianning Wei**

USA

**Shu-Chen Wei**

Taiwan

**Wei Wei**

USA

**Dirk Westermann**

Germany

**Dionna Williams**

USA

**Martin Witzenrath**

Germany

**Chin-Chen Wu**

Taiwan

**Han-Chung Wu**

Taiwan

**Jen-Leih Wu**

Taiwan

**Jinhua Wu**

USA

**Bin-Nan Wu**

Taiwan

**Yi-Chun Wu**

Taiwan

**Hua-Lin Wu**

Taiwan

**Kay LH Wu**

Taiwan

**T.-C. Wu**

USA

**Kou-Juey Wu**

Taiwan

**Betty Wu-Hsieh**

Taiwan

**Xiwen Xiong**

USA

**Jun Xu**

USA

**Yemin Xu**

USA

**Xiaoyu Xue**

USA

**Chuen-Mao Yang**

Taiwan

**Chih-Wei Yang**

Taiwan

**HC Yang**

Taiwan

**Jenq-Lin Yang**

Taiwan

**MH Yang**

Taiwan

**Ruey-Bing Yang**

Taiwan

**Rong-Sen Yang**

Taiwan

**Sung-Sen Yang**

Taiwan

**Shang-Hsun Yang**

Taiwan

**Muh-Hwa Yang**

Taiwan

**Wei-Shiung Yang**

Taiwan

**San Nan Yang**

Taiwan

**Yang Yang**

USA

**Lin Ye**

USA

**Chau-Ting Yeh**

Taiwan

**Wei-Lan Yeh**

Taiwan

**B. Linju Yen**

Taiwan

**Jiin-Cherng Yen**

Taiwan

**Mao-Hsiung Yen**

Taiwan

**Shaw-Fang Yet**

Taiwan

**Hon-Kan Yip**

Taiwan

**Ming-Lung Yu**

Taiwan

**Linda Chia-Hui Yu**

Taiwan

**Wei-Hsuan Yu**

Taiwan

**Dah-Shyong Yu**

Taiwan

**Chiou-Hwa Yuh**

Taiwan

**Shaoyun Zang**

USA

**Ulrich Zanger**

Germany

**Xi Zhan**

USA

**Jing Zhang**

USA

**D Zhang**

USA

